# Outcomes of skin graft reconstructions with the use of Vacuum Assisted Closure (VAC^®^) dressing for irradiated extremity sarcoma defects

**DOI:** 10.1186/1477-7819-5-138

**Published:** 2007-11-29

**Authors:** Alex Senchenkov, Paul M Petty, James Knoetgen, Steven L Moran, Craig H Johnson, Ricky P Clay

**Affiliations:** 1 Head and Neck Oncology Section, University of Cincinnati, 231 Albert B. Sabin Way, Cincinnati, OH, USA; 2 Plastic & Reconstructive Surgery, Mayo Clinic, 200 First Street SW, Rochester, MN 55905, USA; 3 Division of Plastic & Reconstructive Surgery and Department of Orthopedics Mayo Clinic, 200 First Street SW, Rochester, MN 55905, USA

## Abstract

**Background:**

Flaps are currently the predominant method of reconstruction for irradiated wounds. The usefulness of split-thickness skin grafts (STSG) in this setting remains controversial. The purpose of this study is to examine the outcomes of STSGs in conjunction with VAC therapy used in the treatment of irradiated extremity wounds.

**Methods:**

The records of 17 preoperatively radiated patients with extremity sarcomas reconstructed with STSGs in conjunction with VAC^® ^therapy were reviewed regarding details of radiation treatment, wound closure, and outcomes.

**Results:**

STSGs healed without complications (>95% of the graft take) in 12 (71%). Minor loss (6% – 20% surface) was noted in 3 patients (17.6%) and complete loss in 2 (11.7%). Two patients (11.7%) required flap reconstructions and 12 (88%) healed without further operative procedures.

**Conclusion:**

Although flap coverage is an established treatment for radiated wounds, STSG in conjunction with liberal utilization of VAC therapy is an alternative for selected patients where acceptable soft tissue bed is preserved. Healing of the preoperatively radiated wounds can be achieved in the vast majority of such patients with minimal need for additional reconstructive operations.

## Background

Reconstructive surgeons are frequently confronted with irradiated post-ablative skin and soft tissue defects. Muscle and musculocutaneous flaps have been the traditional form of reconstruction in these patients, and little is known about outcomes of split-thickness skin grafts in the setting of preoperative radiation. In some cases, STSG must be considered as the reconstructive option in patients with significant medical comorbidities, recurrence in the area of previous flap, or failed flap reconstruction that is not amenable to microvascular tissue transfer due to lack of recipient vessels. Historically, reported skin graft loss rates in preoperatively irradiated wounds varied from 30% – 100% [1-3].

Modern practice of reconstructive surgery is changing as evidenced by the improvement of surgical techniques, postoperative care, and especially wound care adjuncts. VAC^® ^therapy may simplify reconstruction and improve the outcomes of skin grafts in cases of irradiated defects. The present study was undertaken to evaluate the outcomes of split-thickness skin grafts (STSGs) following oncologic resections in patients with musculoskeletal sarcomas who received preoperative radiation or were treated with locoregional radiation therapy in the past.

## Patients and methods

Retrospective review of the records was conducted to identify the patients who underwent STSG reconstruction of irradiated extremity defects in conjunction with Vacuum Assisted Closure (VAC^®^) therapy. Between January 1997 and December 2005, records of 19 such patients were identified and reviewed with permission of our institutional review board. All patients in this group had soft tissue sarcomas (Table 1). Prior to skin grafting, they were treated with external beam radiation to the tumor bed with the addition of intraoperative radiation or brachytherapy as dictated by treatment protocols.

**Table 1:** Histologic characteristics and distribution of primary tumors

**Histology**	
Malignant fibrous histiocytoma	6
Fibrosarcoma	3
Liposarcoma	3
Angiosarcoma	1
Leiomyosarcoma	1
Synovial	1
Chondrosarcoma soft tissues	1
Fibromyxosarcoma	1

**Tumor location**	

Thigh	8
Lower leg	6
Upper arm	2
Forearm	1

**Total patients**	**17**

All patients had a split thickness skin graft placed on an irradiated recipient bed that otherwise was appropriate for grafting (exposed muscle, vascularized soft tissue, or granulation tissue). The decision to reconstruct with a STSG as opposed to a flap was made by the authors on a case-by-case basis with consideration of the patient's physiological status, oncologic situation, defect characteristics, and patient's and surgeon's preference. The patients that had exposed critical structures such as major nerves, blood vessels, tendons with stripped peritenon, cortical bone, and avascular joint capsule were not suitable candidates for grafting, and no such patients were found in the Mayo Clinic database. Irradiated defects with the exposure of aforementioned structures were appropriately treated with flaps.

**Figure 1:**
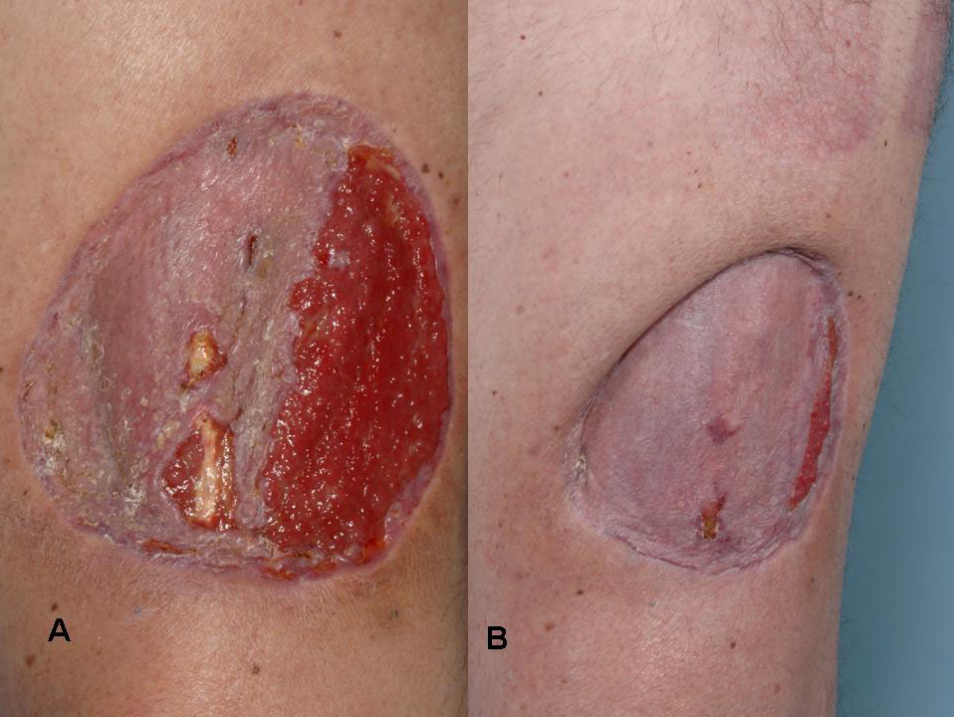
Forty percent graft surface failure of the irradiated defect due to upside down application of the skin graft. Notice prominence of granulation tissue in the irradiated wound in the area of failed skin graft in response to 4 days of VAC therapy (A). Interval healing progress after 7 weeks of local wound care (B).

There were 5 patients with local muscle flaps, which were unequivocally exposed to a full radiation dose. These patients were subsequently reconstructed with STSGs. The patients who had a skin graft applied to an axial pattern muscle flap, which did not have a full exposure to radiation therapy, were not included in the study. Two patients who had skin grafts of irradiated recipient beds were excluded from the study because one had necrosis of the underlying irradiate muscle flap, and the other had a 40% of skin graft placed with epidermis facing the recipient bed (Figure 1). The remaining 17 patients comprised the study group.

Skin grafting was performed as primary reconstruction at the time of tumor resection in 8 patients. Five patients had STSG performed 2 – 8 days following tumor resection as a delayed-primary reconstruction. During this delay, 3 patients underwent a 6-day course of brachytherapy when the brachytherapy afterloading catheters were covered with sterile VAC^®^dressing (Kinetic Concepts, Inc., San Antonio, TX). In the remaining 2 patients, the wound was temporarily covered with VAC^® ^dressing and delayed-primary skin graft was performed within 2 and 8 days following tumor excision when the clear margins of resection were confirmed with permanent pathology. In these 13 patients who underwent primary or delayed-primary skin grafting, the time interval from completion of radiation therapy to skin graft reconstruction varied from 21 to 67 days, average 34 days.

Four patients underwent skin grafting for closure of preoperatively radiated complicated wounds. These wounds were treated with serial débridements and frequent dressing changes, and VAC^® ^therapy. STSGs were used for reconstruction when the wound filled with healthy granulation tissue and was judged amenable to grafting.

All patients underwent reconstruction with 0.012–0.015-inch split-thickness skin grafts that were applied directly on the radiated recipient bed. All skin grafts in this series were meshed 1.5:1 ratio and secured in place with either staples or chromic suture. Xeroform gauze^® ^(Tyco Healthcare Group, Mansfield, MA) or Furacin^®^(Shire US, Inc. Newport, KY) ointment on N-Terface^® ^Interpositional Surface Material (Winfield Laboratories, Inc. Richardson, TX) were placed directly onto the skin graft prior to the VAC^® ^dressing application. Inpatient VAC^® ^therapy at 75 mm Hg in continuous mode was instituted to secure the split-thickness skin graft in place until postoperative day 5. During this time, the patients with lower extremity wounds were kept on bed rest. On the fifth postoperative day, the VAC^® ^dressing was taken down. Following discharge, the patients performed Xeroform gauze^® ^dressing changes once or twice a day for 4 – 6 weeks. All patients had the wounds re-examined postoperatively as a part of their oncologic follow up. Skin graft take was judged by gross inspection, and this information was extracted from the medical records. The end point of the study was complete healing of the wound with stable skin coverage.

## Results

### Patients and oncologic treatments

Skin grafts were performed on 17 patients (9 men and 8 women, age 42 to 82, mean 65). The sizes of skin grafts varied from 23 cm^2 ^to 240 cm^2^, mean 118 cm^2^. All patients had histologically confirmed high-grade (grade 3 or 4) soft tissue sarcomas (Table 1). Two patients had diabetes mellitus, one was a smoker, and none were on steroids. The usual radiation dose was from 50 to 62 Gy with the exception of one patient with a recurrent tumor who received a total of 100-Gy to his recipient bed prior to skin graft reconstruction. On average, the patients received a cumulative dose of 59.3 Gy, ranging from 50 Gy 100 Gy.

### Reconstructive settings

Thirteen patients underwent skin grafting under sterile conditions in the setting of either immediate (primary) or delayed-primary reconstruction. In 4 patients, skin grafts were performed for tertiary intention closure of complicated wounds following surgical site infection and breakdown of primary closure (3 patients) and flap necrosis (1 patient). Thirteen patients had a STSG placed directly on a defect, and 4 patients had it applied to irradiated local muscles flaps (tibialis anterior – 2 and rectus femoris – 2).

### Graft healing

Twelve skin grafts (71%) had greater than 95% graft take and healed completely by primary intention; 3 patients (18%) lost between 6% and 20% of the graft surface; and 2 patients lost their entire graft. Three patients healed by secondary intention: 2 patients with 10% and 20% skin graft loss healed with dressing changes only and 1 patient with complete graft loss healed with wound VAC^® ^therapy. Overall, 15 of 17 patients (88%) healed without further operative intervention, and only 2 patients required reoperation for tertiary intention closure of the defect. One patient with 10% graft loss with exposed tendons of the forearm required a free hemi-latissimus dorsi muscle flap, and the other patient with complete graft loss was salvaged with medial gastrocnemius muscle flaps (Table 2).

**Table 2:** Outcomes of split-thickness skin grafts with VAC utilization in 19 consecutive patients with irradiated extremity wounds

		**Healing of grafted wound (intention)**		
Graft take (%)	Graft (n)	Primary	Secondary	Tertiary (salvage procedures)

95–100	12 (70.6%)	12	-	-
80–94	3 (17.6%)	-	2	1* (free flap)
0%	2(11.7)	-	1**	1 (Gastroc flap)

* Exposed tendons

**VAC therapy only

### VAC therapy and grafting results

VAC^® ^dressing was utilized in all patients to secure the STSG during the early postoperative period. Additionally, VAC^® ^therapy was used for temporary sterile closure of open wounds (5 patients) that allowed delivering brachytherapy (3 patients) and obtaining permanent margins (2 patients) without committing to definitive reconstruction. Four patients with complicated irradiated wounds (3  wound infections and 1 flap loss) were managed with  serial débridements to achieve control of the wound. Until granulation tissue build up was attained, these wounds were treated with VAC^® ^therapy for 16 to 88 days, average 48 days. All 4 patients were skin grafted and successfully healed. Time intervals between skin grafting and completion of preoperative external beam radiation in these patients were 61, 93,123, and 584 days; they had had open wounds for 51, 46, 82, and 16 days, respectively.

One patient deserves a special mention. This patient was originally treated with 45 Gy external beam radiation for primary synovial sarcoma of the lower leg, and the tumor was excised and closed primarily. The patient developed a local recurrence 2 years later and underwent wide-local excision with 35 Gy brachytherapy over the open wound. The defect was reconstructed with a STSG that initially healed, but was lost after receiving a 20-Gy course of additional postoperative external beam radiation therapy to the operated site. At that point, the wound was managed with VAC® therapy for 16 days until it filled with granulation tissue and then was successfully skin grafted after a cumulative radiation dose of 100 Gy.

## Discussion

Split-thickness skin grafts can be used in conjunction with VAC^® ^therapy for reconstruction of irradiated defects with acceptable results following preoperative radiation therapy of soft tissue sarcomas or remote exposure to radiation.

DNA damage is a hallmark of radiation injury to the cell that occurs during radiation therapy. This renders certain susceptible cells, particularly rapidly dividing tumor cells, reproductively incompetent and leads to cell program cell death, apoptosis. While many cells die, those which survive and continue to function have considerably impaired functions and proliferative capacity. This results in compromised wound healing, susceptibility to infections, and marked increase of postoperative wound morbidity [4-6]. These changes however occur in phases. Acute irradiation of the tissues over a short time leads to the initial increase in vascularity that peaks in the second week and then gradually decreases during 4^th ^through 6^th ^weeks as the wound passes the period of subacute inflammation. After the 8^th ^week, vascular density in irradiated wounds becomes lower than it is in controls [7]. These microcirculatory changes are similar to those following radiation therapy and are related to endarteritis obliterans, fibrosis, disseminated thrombosis of the small vessels and chronic ischemia of the tissues [5,8]. Reconstruction is best carried out at the same time or within 4 – 6 weeks of resection, before chronic fibrous reaction sets in [8,9].

Surgery in an irradiated field requires sound clinical judgment. Every plastic surgery technique has been applied to reconstruction of radiated wounds, but there is no simple algorithm in deciding an optimal reconstructive strategy. Although analysis of anatomic characteristics of the defect is guided by general reconstructive principles, the decisions should be made with considerations of extent of radiation damage to the tissues, plans for adjuvant treatments and postoperative surveillance, and functional demands of the patient [2]. Operability and wound healing of an oncologic patient may be affected by malnutrition, anemia, immunosuppression, blood transfusions, chemotherapy, and often age-related medical conditions [10]. Surgical procedures in an irradiated field emphasize sterility to prevent bacterial contamination since these wounds are prone to infection. Meticulous operative technique calls for atraumatic tissue handling, tension-free closure, and obliteration of all dead spaces [6]. Wide, histologically-controlled, negative margins must be assured and, if local control of the tumor is in question, the tumor bed should be readily accessible to surveillance.

Pedicled or free muscular and musculocutaneous flaps allow bringing well-vascularized distant tissue from outside the radiation field that is resilient to both postoperative external beam radiation therapy and brachytherapy. They have an established track record in post-radiation reconstruction because they provide stable coverage, enhance wound healing, and decrease risk of wound breakdowns and infections [3,11]. Microvascular tissue transfer allows the greatest versatility in reconstruction of three-dimensional irradiated defects especially in the head and neck region. They can be safely anastomosed with irradiated vessels of the recipient site [12,13]. Distant non-irradiated pedicle flaps are a great asset, but within the radiated field they can be treacherous, and their use in chest wall reconstruction resulted in 32% wound complication rate and 14% total flap loss [14]. One of five irradiated local muscle flaps was lost in the present study, leading to the complete failure of the skin graft and an amputation of the extremity.

Split-thickness skin grafting is simple to perform and has a low morbidity. If final margins are found positive, prompt re-excision can be performed without the need of excision of the flap along with all the tissue planes of dissection and surgical drain tracks that were intraoperatively seeded with tumor. It facilitates oncologic surveillance of the tumors with a high rate of local recurrence, such as many soft tissue sarcomas. Concerns have been voiced regarding the use of STSGs in the setting of preoperative and postoperative radiation therapy, and this issue remains controversial. Animal data suggested that STSGs were vulnerable to adjuvant radiation and tolerated doses within only 25 Gy limit [15]. On the other hand, 90% of patients retained stability of their wound coverage with STSG following adjuvant radiation (59 ± 0.9 Gy) in the study from Memorial Sloan-Kettering Cancer Center [16].

There is no agreement about the use of STSGs for reconstruction of irradiated wounds. High failure rates without clear correlation with the radiation doses were historically reported in the literature [1-3] and no clear data on skin graft outcomes in irradiated wounds have been published to date. Because skin graft take is largely dependent on inosculation and neovascularization, failure of the STSG in an irradiated field was ascribed to vascular phenomena [9,17]. Changes in the vascular bed and fibrosis associated with the ensuing chronic phase of radiation insult negatively influence the result of reconstructive operations. While in mild-to-moderate radiation impairment of the tissues, skin grafting could be considered [6], the success of skin grafts in an irradiated field is unpredictable. Rudolph reported 100% skin graft loss requiring reoperation regardless whether or not irradiated wounds were excised [3].

Laboratory and clinical studies have shown that the VAC^® ^therapy increases wound blood flow, granulation tissue formation, and decreases accumulation of fluid and bacteria [18]. Recently, conformational changes in the cytoskeleton of the cells in response to application of micromechanical forces, i.e. stretch of the wound surface by the irregularity of the VAC sponge, was postulated to be an important factor in VAC-augmented wound healing [19]. Similar mechanism of stretch-induced cell proliferation is thought to be the driving force in tissue expansion [20,18,21] and distraction osteogenesis [22,23]. Initially applied for treatment of the chronic wounds, it was found to be useful in the management of acute wounds as well. VAC^® ^therapy has been shown to hasten wound closure and the formation of granulation tissue in a variety of settings [24-26].

VAC^® ^dressings are successfully used for securing skin grafts [24,25,27], especially in wounds with exudative, irregular, or mobile recipient beds and in difficult anatomic locations [25,28-30]. The manufacturer guidelines recommend continuous mode negative pressure of 75 mm Hg to 125 mm Hg [31]. We traditionally used 75 mm Hg for skin graft application although 125 mmHg negative pressure has also been used by other authors [25,27,32,33]. The VAC^® ^stabilizes the skin graft and conforms well to the shape of recipient bed, removes fluid, decreases bacterial counts, and provides a secured dressing [27]. Improved graft survival and reduced need for repeat skin grafting were noted in one retrospective study [32].

The function of the VAC^® ^technique in irradiated wounds is largely unknown and clinical experience is very limited [34]. In the present study, VAC^® ^technique was used in the vast majority of patients in four types of settings: coverage of afterloading catheters during brachytherapy, optimization of the wound prior to grafting, securing the split-thickness skin graft, and secondary closure of skin graft losses.

Immediate flap reconstruction has been traditionally advocated for coverage of afterloading brachytherapy catheters [35,36]. Utilization of the VAC^® ^in lieu of immediate flap reconstruction in sarcoma patients requiring brachytherapy provided rapid and simple temporary coverage with good stability of the catheters. It allows sparing the flap form radiation insult and performing an elective delayed reconstruction after completion of brachytherapy [30]. Patients who underwent flap reconstructions were not included in this series. However, further study is indicated concerning the effects of temporary VAC^® ^coverage on brachytherapy effectiveness. Theoretically, the increased blood flow and oxygenation may minimize postoperative tissue ischemia and improve effectiveness of brachytherapy.

VAC^® ^therapy was also used for preoperative optimization of irradiated wounds to allow skin grafting of a viable, granulating bed as well as secondary closure of the skin graft breakdowns. Radiation primarily impairs small vessels, decreases their size and density [17], which causes local hypoxemia, decreased bacterial clearance, and impaired regeneration, but the VAC^® ^counteracts all these effects. It quadruples local blood flow, promotes delivery of oxygen and nutrients, decreases bacterial counts, increases size and density of capillaries, and promotes the growth of granulation tissue [18,24,37,38]. Successful skin graft take was achieved in one patient who received a cumulative local dose of 100 Gy as a result of VAC^® ^utilization for optimization of the wound and graft application.

The present study retrospectively analyzed a group of extremity sarcoma patients who received compatible doses of preoperative radiation and had a close follow up. These data give insight into the clinical behavior of STSGs following standard doses of preoperative radiation therapy commonly used in soft tissue sarcoma treatment. Despite small breakdowns of the skin grafts that eventually healed and are common in non-irradiated grafts, only 2 patients (11.7%) had complete graft loss. Eighty-eight percent of patients in this study eventually healed without requiring further operative procedures. The data demonstrate that the reconstruction of the suitable irradiated soft tissue wounds with STSG in conjunction with VAC^® ^therapy may be considered as an acceptable reconstructive approach in the setting of questionable margins, high risk of recurrence, and poor physiological condition of the patient.

## Conclusion

Split-thickness skin grafting provides a simple one-stage reconstructive option for skin and soft tissue defects, but its use in the irradiated wound is controversial. In the present study of skin-grafting of post-ablative defects in the preoperatively radiated soft tissue sarcoma patients, we found that with meticulous surgical technique and utilization of VAC^® ^therapy complete or partial skin graft take and complete healing of the wound without reoperation was achieved in 88% of cases. Complete (>95%) skin graft take was observed in 71% of cases, partial (80%–94%) take in 18%, and complete loss of the graft in 12%. Only 12% of skin-grafted wounds required closure with an additional operation. Split-thickness skin graft reconstruction of irradiated skin defects can be performed following preoperative radiation therapy of soft tissue sarcoma patients in conjunction with VAC^® ^therapy with acceptable skin graft take rate and minimal morbidity to the patients.

## Abbreviations

STSG: split-thickness skin graft

VAC: vacuum-assisted closure

Gastroc flap: gastrocnemius muscle flap

## Competing interests

The author(s) declare that they have no competing interests.

## Authors' contributions

AS: conception and design, acquisition of data, analysis and interpretation of data, drafting the manuscript, revising it critically for important intellectual content

PMP, JK, SLM, CHJ and RCP: conception and design, analysis and interpretation of data, revising the manuscript critically for important intellectual content
